# Determining decoating efficiency for mechanically stressed catalyst coated membranes of proton exchange membrane water electrolysers

**DOI:** 10.1111/jmi.70000

**Published:** 2025-06-16

**Authors:** Malena Staudacher, Andréa de Lima Ribeiro, Ruben Wagner, Margret Fuchs, Anja Weidner, Thomas Buchwald, Urs A. Peuker

**Affiliations:** ^1^ TU Bergakademie Freiberg Institute for Mechanical Process Engineering and Mineral Processing Freiberg Germany; ^2^ Helmholtz Institute Freiberg for Resource Technology Freiberg Germany; ^3^ TU Bergakademie Freiberg Institute of Materials Engineering Freiberg Germany

**Keywords:** µXRF, decoating, image processing, mechanical processes, PEMWE, recycling, SEM

## Abstract

The recovery of critical raw materials from water electrolysers, which are used to produce green hydrogen, is essential to keep the raw materials with limited availability in the material cycle and to facilitate the expansion of production of this technology, which is supposed to be essential for the decarbonisation of our industrial society. Proton exchange membrane water electrolysers (PEMWE) use precious metals such as Ir and Pt as catalysts, which require a high recycling rate due to their natural scarcity. In order to investigate at an early‐stage mechanical recycling technologies, such as shredding for liberation and milling for decoating of these complex materials, it becomes necessary to develop small‐scale experimental methods. This is due to the low availability of End‐of‐Life samples and the high price of pristine electrolyser components. Especially decoating has shown huge potential for a highly selective separation of defined material layers; nevertheless, until now, there is no method to determine the success of decoating of the flexible polymer membrane, which is coated on both sides with particle‐based electrodes. One possible concept is presented here, using scanning electron microscope images and micro‐X‐ray fluorescence elemental maps. Image processing and segmentation is performed using the WEKA software and a simple thresholding method. This allows the efficiency of the decoating process to be determined with an accuracy of ±0.5 percentage points for decoated PEMWE cell samples. The high accuracy of the presented method framework provides the necessary tool for any further quantitative development of improved mechanical stressing for decoating.

## LAY DESCRIPTION

It is important to recover raw materials from water electrolysers that produce green hydrogen. This helps to keep the raw materials in the material cycle and allows electrolyser production to be increased. Polymer electrolyte membrane water electrolysers require precious metals as catalysts. However, these are in short supply and must therefore be recovered. In order to investigate whether the catalysts can be detached from the membrane, methods must first be developed that work on a small scale. So far, there is no method to determine whether the flexible polymer membrane has been successfully decoated. The concept presented here uses imaging methods to visualise the decoating. The images are then digitally processed. This allows the efficiency of the decoating process to be determined with an accuracy of ±0.5 percentage points for partially decoated electrolyser cell samples. This is important for improving mechanical processes like decoating.

## 1 INTRODUCTION

Water electrolyser technologies are an important part of the production of green hydrogen. The target of the German government's national hydrogen strategy of 5 GW electrolyser capacity by 2030^1^ was doubled to 10 GW^2^ in the progress report, which will generate an enormous demand for electrolysers. Proton exchange membrane (PEM) electrolysis is an emerging technology that stands for high current densities, high cell voltage efficiencies and can adapt to renewable energy sources due to its fast system response.^3^ According to the EU report^4^ on critical raw materials, platinum group metals (PGMs), which are extremely important for PEM electrolysis, are among the materials with the highest supply risk due to their limited availability and market concentration^5^ of the supplying countries. They have been classified as even more critical from 2020 to 2023 and this trend will continue in the upcoming years. This low availability of key precious metals imposes a challenge for scaling up the production of PEM water electrolysers (PEMWE).^6^ To secure the supply, a high recycling rate of at least 90% is essential, in particular of Ir catalysts^5^ due to material prices and value associated to it. Due to the economic aspects, industry is aiming for even higher recycling rates of at least 99%.

An additional challenge is identified in the material supply logistics, which is the lifetime of PEMWE stacks. These vary from 50,000 to 80,000 operation h^7^, ^8^, ^9^ corresponding to an average usage of 7–10 years.^10^, ^11^ This puts further pressure on the development of methods to enable high recycling rates. It is imperative for a secure and sustainable supply of raw materials that the critical elements are recycled as completely as possible to be available for new electrolysers. Raw material supply and recycling are also important challenge for the management of lithium‐ion battery waste and have been subject of numerous studies over the years.^12^, ^13^, ^14^ For comparison, the lifetime of PEMWE is even shorter than in lithium‐ion batteries (10–15 years^15^, ^16^), resulting in faster material cycles and an increasing need for recycling. This, together with the increasing demand for electrolysers to meet governmental development targets, is leading to an intensified research focus on PEMWE recycling.

Since the electrolyser system with Balance of Plant and Power Electronics has a life expectancy of about 20 years^10^, ^11^ and recycling of those components is state of the art,^17^ the focus of PEMWE recycling is on the electrolyser stack. Components such as bipolar plates and end plates can potentially be refurbished and reused,^17^ which is being investigated for fuel cell stacks in the ReStack^18^ and Stack2P^19^ projects, while the electrochemical cell, which is usually decisive for the end of lifetime,^20^ cannot be reused but has to be recycled.

For this reason, and because critical precious metals such as Pt and Ir are used as catalyst particles in the electrochemical cell of these electrolysers, the focus of this investigation is on the cell. The catalysts are applied as composite coatings to both sides of the name‐giving proton exchange membrane (PEM), also called polymer electrolyte membrane, in layers with thicknesses in the single digit micrometre range.^21^ This layered composite is called catalyst‐coated membrane (CCM).

In addition to the conventional recycling strategies for CCM, such as pyrometallurgy, other approaches are currently under investigation. These include the complete dissolution of the CCM in a solvent or the targeted decoating of the membrane from the electrodes. The latter method has been shown to leave the membrane largely undamaged. The extant literature has so far documented the use of solvent decoating; nevertheless, it is also conceivable to employ dry mechanical stress. This method has been shown to allow targeted decoating of the layers without complete comminution for liberation of the material. In order to recover the valuable materials, the electrolytic cell must first be demounted from the electrolyser stack, which can be achieved by (automated) disassembly.^22^ Once the cell is removed, the valuable electrode layers can be decoated and recovered by mechanical methods.

Composite materials for new applications, which are still in development, are available only in limited quantity for research. This also applies to PEMWE CCM: too little End‐of‐Life (EoL) materials are available, which are needed for recycling studies. Due to the high price of virgin materials and the small quantities available, recycling has to be studied on very small sample masses, that is, in the range of 100 mg and less. As a result, the choice of mechanical processing equipment suitable for this comminution task is restricted. In addition to comminution, the analytical methods tailored to assess the success of mechanical recycling on the mg scale are also very limited. An important objective is therefore to find a method that can quantitatively determine the success of decoating for the particular layer structure of CCM at very low sample masses.

Parameters used to assess the degree of decoating were studied for lithium‐ion batteries and comprise basically mass balance. Thus, Wuschke,^23^ Werner et al.^24^ and Lyon et al.^25^ have defined a mass‐specific degree of decoating for electrode foils. They have used the ratio of decoated mass to the total mass of coating, which itself is the sum of the coatings on both conductive battery foils. The mass of the entire coating was calculated by manual disassembly and subsequent identical analysis for the graphite containing anode and the metal mixed oxide containing cathode. Other methods of describing lithium‐ion battery decoating are called compound separation efficiency^26^ and peel‐off efficiency,^27^ but are based on the same approach of using the mass of the coating. This concept for lithium‐ion batteries cannot be transferred to the electrode product of PEMWE, because the total mass of the electrodes processed per experiment is significantly lower than that for lithium‐ion battery samples, which means that potential mass losses have a greater influence and lead to potentially significant systematic errors. In addition, due to the early development stage the electrode mass is different for each CCM. Furthermore, the precious metal content of the electrodes cannot be determined because it is an EoL product where the catalysts dissolve and migrate into the membrane^28^ and therefore the precious metal content is not characteristic for the coating only.

Kaiser et al.^29^ used high‐intensity cavitating ultrasound to remove the coating from solid oxide cells in high‐temperature electrolysers. The success of the decoating process was determined by comparing the area of the decoated cell to the diameter of the sonotrode. The decoated area was determined using digital images and subsequent digital image processing. Kaiser et al.^29^ were able to use a simple thresholding method due to the significant colour (grey scale) difference of coated and decoated areas of the cell. However, since this colour difference is not visible in all cell types, the authors had to perform a contour extraction or manual segmentation for the remaining cells, which did not show that colour feature.

An alternative approach to quantify the removal of the electrode layer is the analysis of the remaining membrane pieces or the membrane surface. Danerol et al.^30^ have documented the decoating progress of PEM fuel cell (PEMFC) CCM when exposed to ultrasound using an optical microscope in transmission mode. Since this method can only monitor the decoating when the respective coating layers are removed on both sides of the electrode, it is not possible to resolve the individual, side‐specific degrees of decoating. The optical microscope was also used for the evaluation of a peel test.^30^ The peeled tape with the electrode fragments was inspected and visually evaluated, again based on colour. Defects in the electrode, adhesive failure at the phase boundary between membrane and electrode, and cohesive failure in the electrode layer could be distinguished.

A novel methodology for determining the success of decoating for PEMWE CCM based on a surface measurement analysis is proposed here. Methods such as scanning electron microscope or micro‐X‐ray fluorescence generate an image or map of the sample surface, in which the coated and decoated areas are distinguishable based, for example, on the detection of dominant chemical elements. The elements differ depending on whether the electrode is still present or has been removed and the membrane underneath is visible. Additional challenges in the analysis of the PEMWE CCM decoating success by such method include the properties of the membrane material, that is, the flexible, non‐planar nature of the polymer membrane, the spatial resolution in the lower micrometre range (for sample sizes of a few millimetres) of the element sensitive imaging method, and the need for a non‐destructive and non‐adhesive measurement, since both sides of the membrane are of interest.

Considering these criteria, an investigation on three potential methods suitable for the task is presented: scanning electron microscope (SEM), energy dispersive X‐ray spectroscopy (EDS) and micro‐X‐ray fluorescence (µXRF). Both experimental material and mechanical decoating processes are described, as they are essential for understanding the quantification results. Finally, the challenges associated with image processing and the additional steps required for determining the success of the decoating process are discussed.

## 2 MATERIALS AND SAMPLE PREPARATION

The cell pieces investigated here were produced during the mechanical stressing process in a small‐scale hammer mill typically used in pharmaceutical research.^31^, ^32^ Since CCM from electrolysers in particular have low availability and high prices, membrane electrode assemblies (MEA) from PEMFC stacks were used. The FC CCM is very similar in the structure to cells from an water electrolyser (WE).^33^ The only differences are the thinner membrane (about 50–250 µm for WE and 10–50 µm for FC^34^) and the use of Pt instead of Ir as catalyst in the anode electrode.^35^ Fuel cells can be used to perform initial tests on the polymeric membrane and to develop the analytical method for decoating assessment and estimation. This will provide important insights that can be applied to the recycling of electrolyser cells. The larger membrane thickness of the PEMWE ensures, that if the membrane remains intact for PEMFC it will as well for PEMWE, thus similar samples can be expected.

**FIGURE 1:**
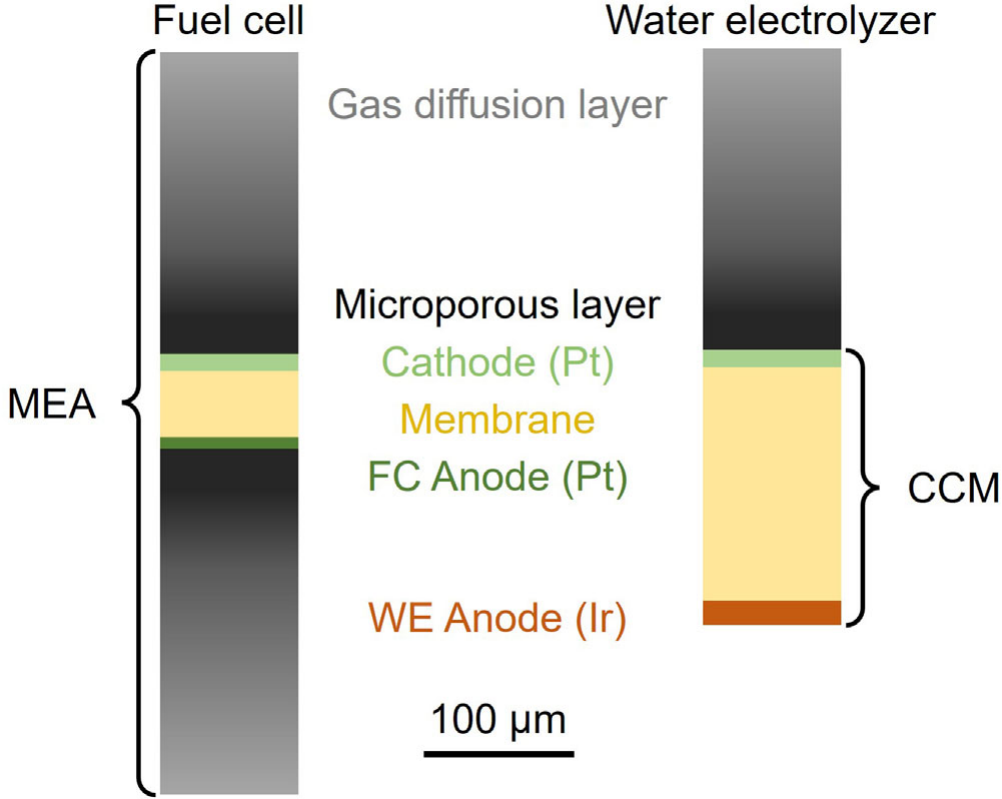
Schematic of fuel cell and water electrolyser MEA, highlighting similarities and differences.

The material utilised in this investigation originated from a 40‐cell FC stack with 4000 operating hours (see Al Assadi et al.^22^ stack 1), which was first disassembled manually in order to have the individual components. The 7‐layer membrane electrode assembly (MEA) consists of a polymer electrolyte membrane made of perfluorosulfonic acid (PFSA) coated on both sides with the electrode material, which together form the CCM. The electrode material consists of carbon black particles doped with a Pt or Pt/Ru catalysts and a suitable polymeric binder system.^22^ A microporous layer (MPL) made of carbon is deposited on the electrode layers, followed by a gas diffusion layer (GDL) of carbon fibres. A schematic representation of the FC MEA is shown in Figure 1 in comparison to a typical WE MEA. An representative SEM image of the FC MEA used here can be found in Al Assadi et al.^22^

**FIGURE 2:**
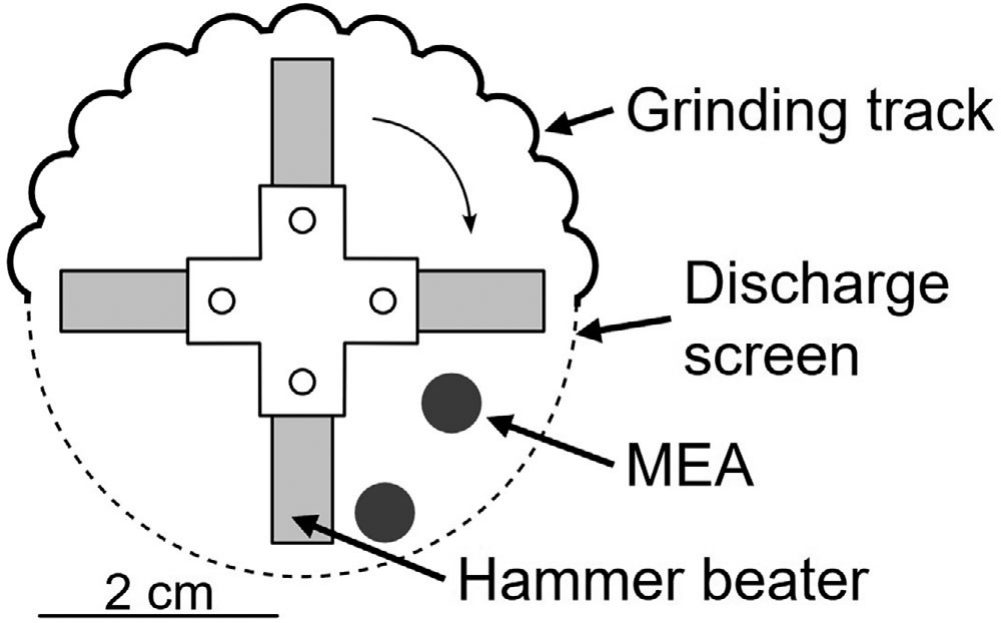
Schematic of laboratory hammer mill Picocrush with spherical MEA samples (discs).

Ten circular samples with a diameter of 5.6 mm were punched from the described MEA as uniform feed material for the decoating experiments. The sample disks were comminuted in the laboratory hammer mill Picocrush (Figure 2) on the machine platform Picoline (Hosokawa Alpine) for 30 s at varying rotor speeds (5000 or 7000 rpm). The discharge screen of the mill has circular holes with a diameter of 1 mm. The fine comminution product that passed the screen consists of carbon fibres from the GDL, carbon particles from MPL and decoated electrode powder. The coarse product consists of the large decoated membrane pieces that are the subject of this study. In most experiments the underlying membrane of the circular MEA samples did not fracture and kept its size and shape.

**FIGURE 3:**
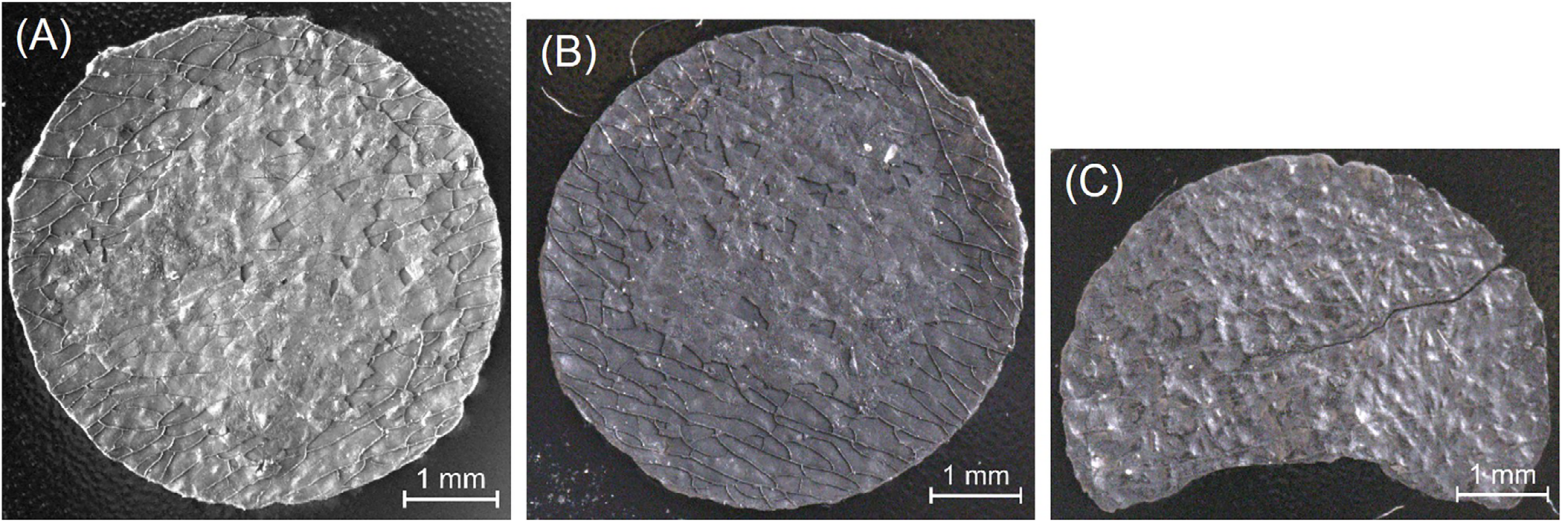
Digital images of selected samples from decoating experiments in the hammer mill (A) sample 5000/1; (B) sample 5000/2; (C) sample 7000/1.

Three samples of the treated MEA pieces were randomly selected from the experiments: two samples from the experiment at 5000 rpm with the cathode side up (5000/1 and 5000/2) and one sample treated at 7000 rpm with the anode side up (7000/1) (Figure 3). These samples are glued to SEM specimen holders with carbon adhesive tape, allowing one side of the cell to be examined for state of decoating. This specimen holder with the samples was used for all imaging techniques.

## 3 METHODS

### 3.1 Imaging techniques

#### 3.1.1 Micro‐X‐ray fluorescence

Micro‐X‐ray fluorescence (µXRF) is a technique suitable for identification of elemental composition at spatial resolutions in the micrometre range. The Bruker M4+ Tornado XRF spectrometer (Helmholtz Institute Freiberg for Resource Technology) was employed for the generation of µXRF‐based elemental maps of the selected CCM pieces (Figure 3).

The equipment uses a Rhodium anode, which generates an excitation beam with diameter of 20 µm (50 kV, 600 µA = 30 W of total power). The excitation beam is focussed onto the cell surface by polycapillary lenses and the µXRF is acquired for 50 ms per point, that is, per pixel in the elemental maps. The distance between acquisition points, that is, the spatial resolution of the elemental maps, was chosen as 20 µm (125 s/mm^2^), which is the smallest spot size possible on the Bruker instrument, and 50 µm (20 s/mm^2^). For the analysis of one sample (6 × 6 mm^2^), the measurement time is reduced by a factor of 6 from 75 min to approx. 12 min.

To determine the areas of the membrane that have been decoated and thus exposed, an appropriate elemental map must be selected. The membrane consists of PFSA, which contains C, O, H, F, and S. Since µXRF detects elements beginning with Na (atomic number *Z* = 11), S (*Z* = 16) and not F (*Z* = 9), is used here as an indicator of the decoated membrane. Since Pt is the catalyst in the electrode of fuel cells, Pt is used as an indicator for the coated areas from the numerous elemental maps obtained, assuming that Pt is uniformly distributed within the coating. However, if the selected maps are viewed individually, over‐ or underestimation of electrode removal efficiency can occur due to blurred transitions caused by the partial area effect. It is not possible to determine exactly where the areas to be segmented begin when looking at only one element. By using both elements for the membrane and the electrode, the transition can be defined more precisely. Therefore, the two maps were merged as described in Section 3.2.

Considering that the membrane matrix consists of PFSA (CHF_7_O_3_S, density of 2 g/cm^3^) it is estimated that 95% of signals associated with Pt were collected from a depth of up to 379 µm (information depth from BRUKER GmbH calculation software, pressure = 2 mBar). Both detection and generation of elemental maps were performed by the software provided with the measuring device.

The elemental maps correspond to spatially resolved signal acquisitions of a single element, represented as grey‐scale images. In these images, the maximum signal intensity for a given element results in a pure‐white pixel (R: 255, G: 255, B: 255) whilst the lowest signal intensity is represented by a pure‐black pixel (R: 0, G: 0, B: 0).

#### 3.1.2 Scanning electron microscopy

The cell pieces fixed with carbon adhesive tape were investigated with a field‐emission scanning electron microscope (SEM; MIRA 3 XMU, Tescan, Czech Republic) at the Institute of Materials Engineering at TU Freiberg with an acceleration voltage of 25 kV. To distinguish coated from decoated areas, backscattered electron (BSE) contrast was used (working distance: ∼15 mm) to obtain overview images of cell pieces. The noble metal containing electrode (coating layer) exhibits reflection signals of higher intensity than the organic polymer membrane below. Consequently, the brighter values enable to distinguish the respective areas and determine the decoating result. Additionally, energy‐dispersive X‐ray spectroscopy (EDS) at an acceleration voltage of 20 kV and a dwell time of 50 µs was used for elemental mappings of the respective cell pieces as a further analysis method. The pixel size of the elemental maps is around 20 µm. Unlike µXRF mapping, the target element F can be detected and is therefore chosen as the indicator for the PFSA membrane as it is the most abundant and detectable element in the membrane. Pt is again used as an indicator for the electrode.

### 3.2 Image processing and segmentation

After image acquisition, the decoated areas of the membrane are clearly visible in the elemental maps of the µXRF and EDS and data sets of SEM‐BSE. To elaborate the decoating quantitatively, the images were digitally processed. The goal is to determine the decoating efficiency, which is defined here in 2D as the ratio of the decoated area *A*_decoated_ to the total area of the sample *A*_total_ (see Equation 1). Each image consists of background information not associated with the cell material, coated sample areas, and decoated sample areas. In order to clearly distinguish coated and uncoated regions, the image pixels correspondent to the background has to be excluded (image segmentation). After segmentation, a false colour image must be generated for image processing, from which the pixel number *n* of the segmented areas can be determined to calculate the decoating efficiency *E*.

(1) $$E=\frac{A_{\mathrm{decoated}}}{A_{\mathrm{total}}}=\frac{n_{\mathrm{decoated}}}{n_{\mathrm{total}}}.$$

In order to compare the accuracy of the individual methods with each other, a relative error *F*_rel_ is calculated for each segmentation method *i* and imaging method *j*, which indicates the deviation of the calculated decoating efficiency from a reference value (see Equation 2). As a reference, the mean value of the manual segmentation (*i* = Manual) for each imaging method is used. The individual segmentation methods are discussed in detail below.

(2) $$F_{\mathrm{rel}}=\frac{E_{i,j}-\bar{E_{Manual,j}}}{\bar{E_{Manual,j}}}.$$

To ensure that the image processing routine was performed identically for all images, automated routines in Python programming language (version 3.10.9) were developed for data evaluation, colouring and simple thresholding. In addition, ImageJ (version 1.53q) was used to perform the manual processing, which mainly includes trainable WEKA (Waikato Environment for Knowledge Analysis) segmentation and manual marking. The Python code is provided as supplementary material.

**FIGURE 4:**
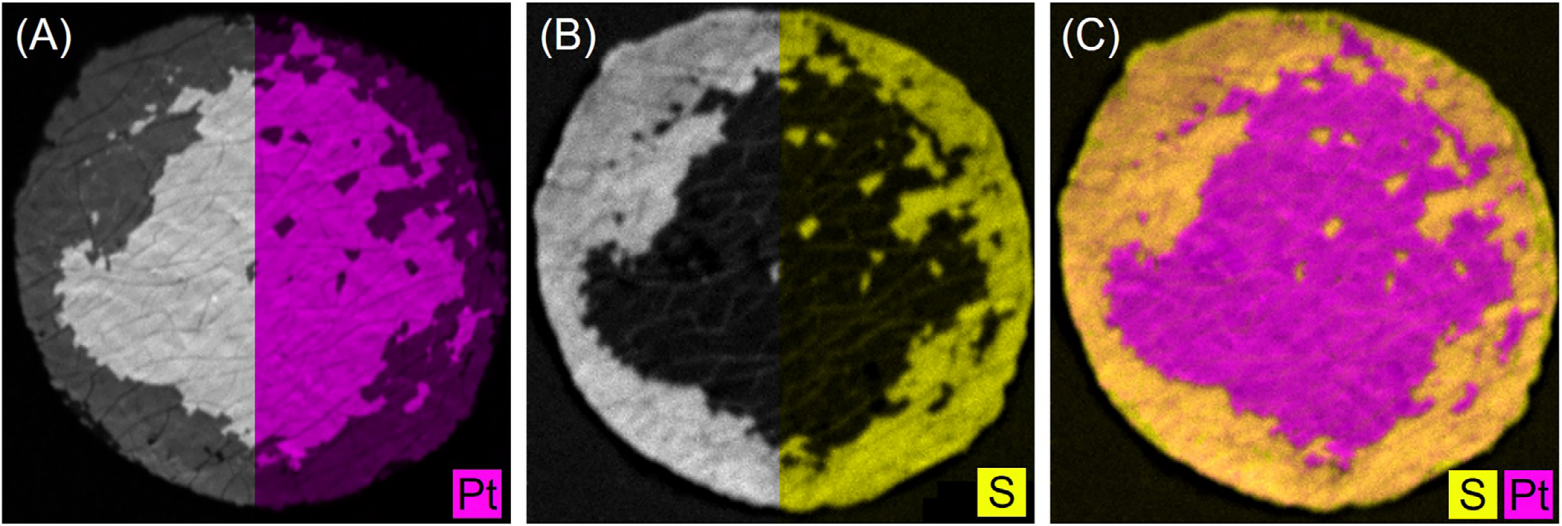
Illustration of the image processing steps for µXRF data using sample 5000/1 as example: (A) left: µXRF grey‐scale map for Pt, right: coloured with hue = 212.5 for Pt; (B) left: µXRF grey‐scale map for S, right: coloured with hue = 42.5 for S; (C) merged coloured µXRF maps (A) and (B) for elements Pt and S.

Prior to image segmentation, all images were preprocessed to mitigate the occurrence of erroneous segmentation. Firstly, scale bars and background irregularities were manually removed. In addition, the grey‐scale element maps from µXRF and EDS were coloured by assigning an element‐specific hue in the HSV colour space (212.5 for Pt, 42.5 for S and 85 for F in EDS images), using the existing grey scale as the value. The saturation is set to 255 for these coloured images. Figure 4A and B shows the elemental maps for Pt and S as a grey value image on the left and coloured with the element‐specific hue on the right.

Since the grey values in the S‐map do not clearly indicate what is background and where the membrane is still coated, the information from S and Pt maps must be merged. This is the only way to create a meaningful image for unambiguous segmentation. Therefore, the coloured elemental maps are overlaid with Python, whereby the blending factor of both maps is set to 1 in order to obtain strong contrasts and colours. This results in two‐colour images shown in Figure 4C and is used in the following.

Note that this naturally results in a colour transition between the decoated membrane and the electrode, which is highly dependent on the previously selected element‐specific hue. The hue values have been chosen so that the colours are as far apart as possible in the colour wheel so that they can be easily distinguished. However, the merging of the images, the choice of colours, and the method of merging the images introduces uncertainties in the determination of the individual areas.

#### 3.2.1 Manual segmentation

**FIGURE 5:**
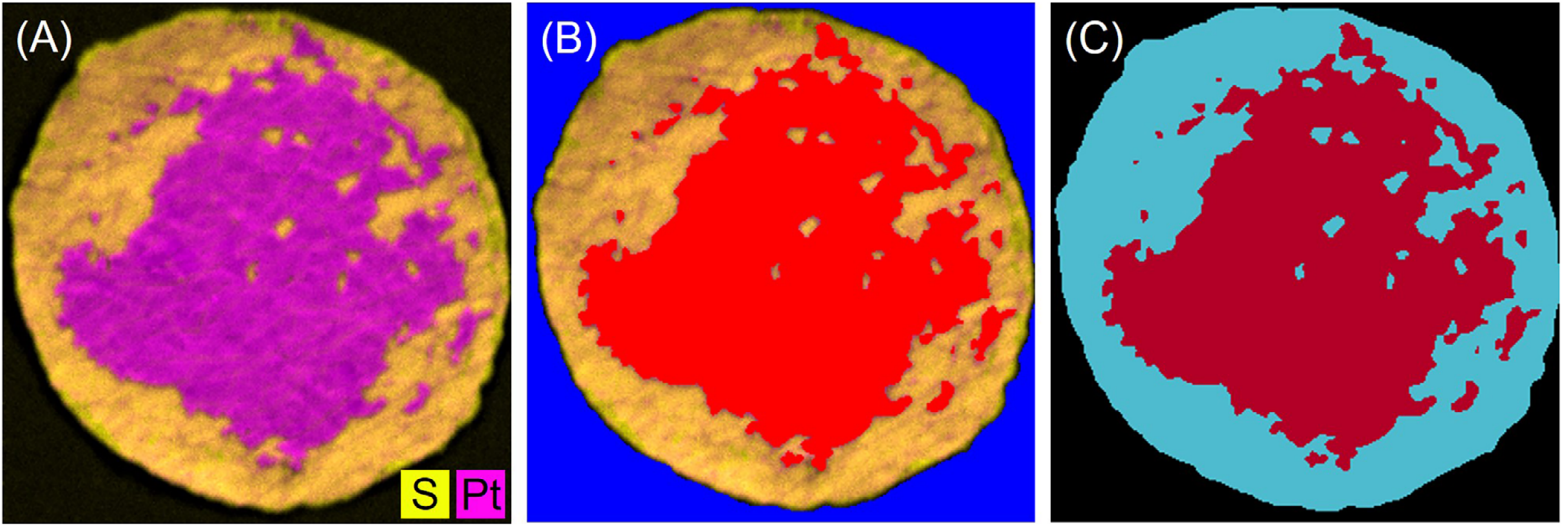
Manual segmentation steps using sample 5000/1 as example: (A) µXRF merged elemental maps (Pt and S); (B) manually delimited electrode (red) and background (blue) regions; (C) generated false colour image of the circular sample (red—electrode; blue—decoated membrane).

In order to have reference values for the decoating efficiency, the images of all analysis methods were manually segmented. The electrode areas were outlined with the ImageJ software using freehand selection, labelled as electrode and filled with a specific colour for visualisation. In addition, the membrane outline was then traced, so the background was labelled and coloured differently (Figure 5B). An in‐house developed workflow in Python was used to create masks for each colour and merge them to generate a false colour image which highlights the characteristics of the membrane under analysis (Figure 5C). In the resulting false colour images, red always indicates coated electrode and blue the decoated area. The decoating efficiency can then be calculated according to Equation (1). This procedure was performed three to five times for each image to ensure reproducibility and to estimate the potential scattering.

#### 3.2.2 Thresholding

**FIGURE 6:**
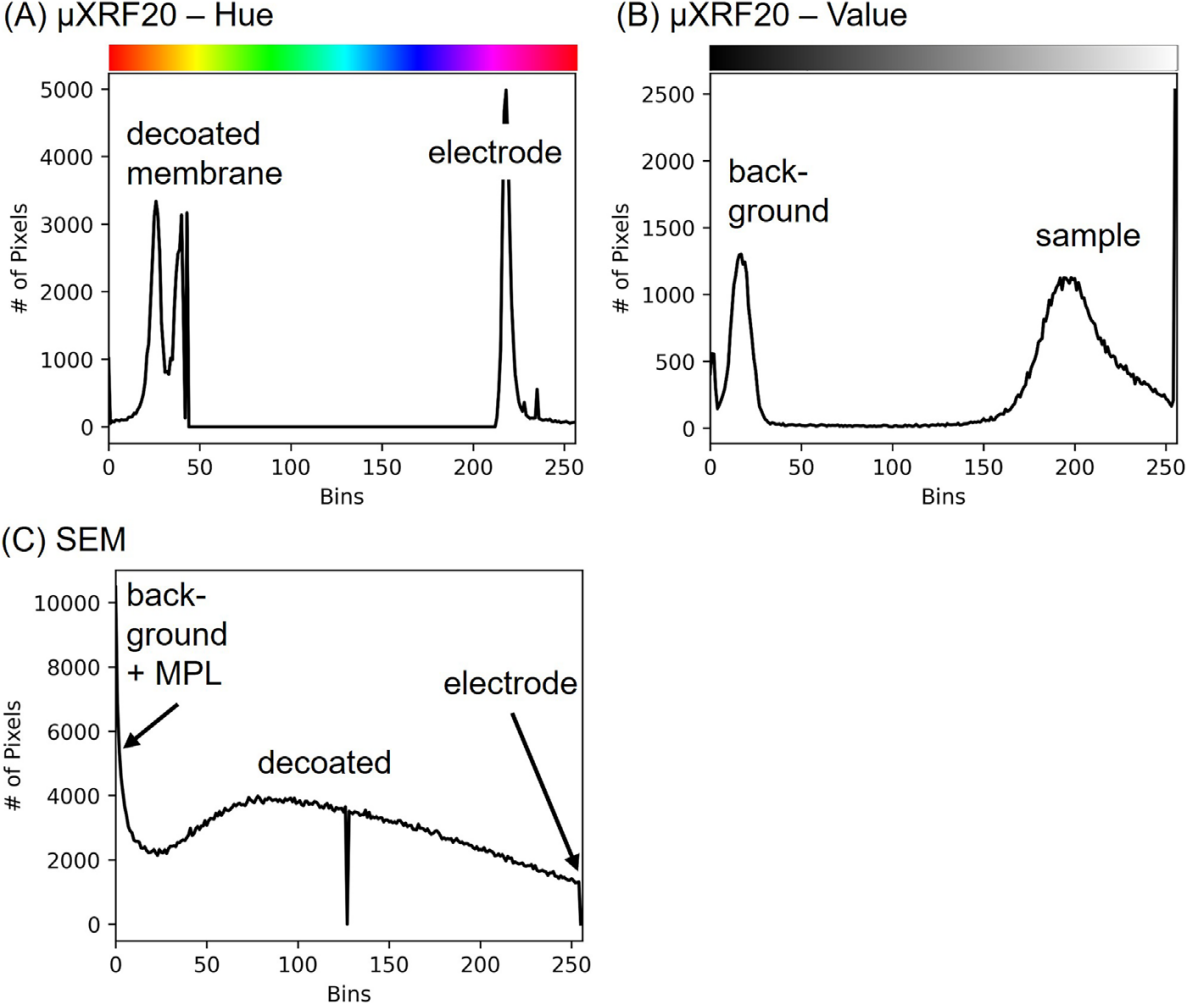
Selected parameters and their value distribution of sample 5000/1 used for thresholding (A) Hue histogram from merged µXRF map. (B) Value histogram from merged µXRF map. (C) histogram of SEM image.

The thresholding had to be adapted for each type of data set, as the images and maps varied according to the technique employed. The µXRF images were segmented using a colour threshold in the HSV colour space, since a distinct bimodal distribution of the hue value could be generated by element‐specific colouring as described in Section 3.2 (Figure 6A). While the segmentation between the hue values 50 and 200 is very clear because there are no values in this range, the transition between the two areas can be seen in the colour gradient between the values 230 and 20 (in the red range). Since the hue values are a colour wheel and not a spectrum like a grey scale, we need to define not one, but two boundaries for the segmentation; here, the second boundary is drawn at the transition from 0 to 255.

**FIGURE 7:**
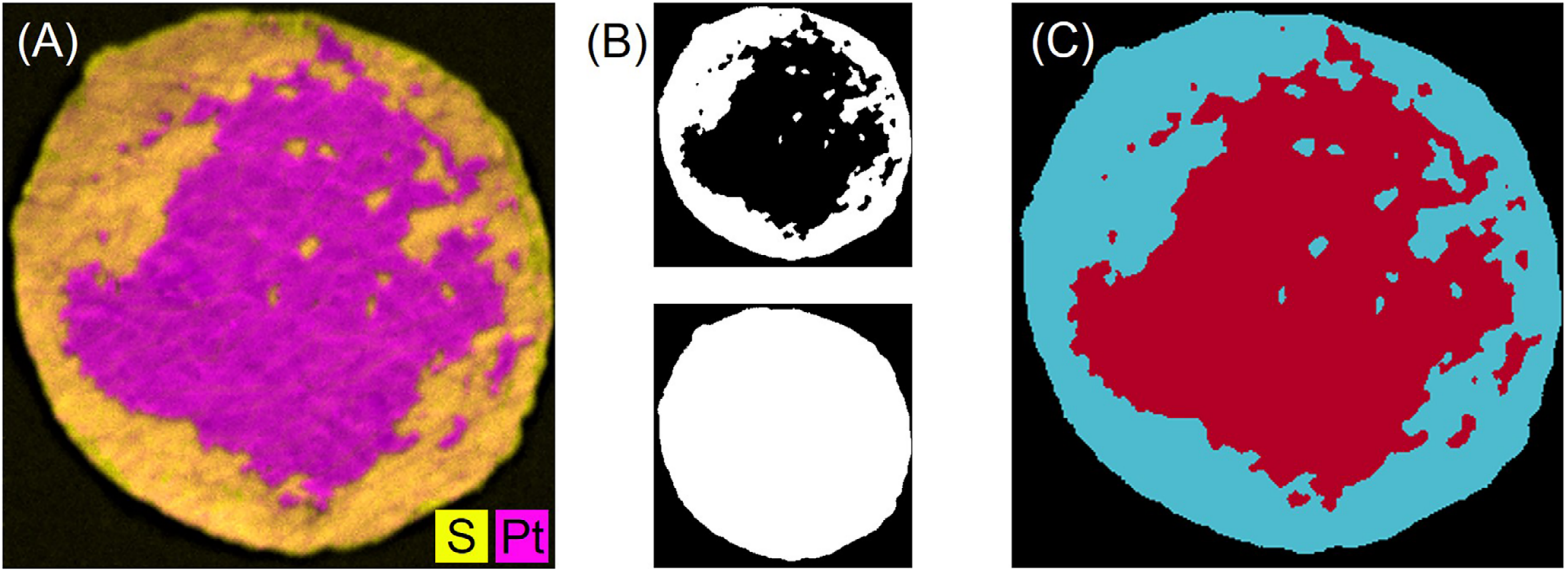
(A) µXRF merged elemental maps (Pt and S). (B) Binary masks obtained from HSV thresholding method. (C) Generated false colour image of the circular sample (red—electrode; blue—decoated membrane).

In Figure 6B a bimodal value distribution of the grey scale for background pixels can be observed, which is the main criteria for reliable identification of the sample's edges using Otsu's method. Otsu's method assumes that the original image contains pixels from two classes.^36^, ^37^ In Figure 7, the original image (Figure 7A), the two binary masks (Figure 7B) and the resulting false colour image (Figure 7C) are presented.

Thresholding of SEM images is more demanding than for µXRF images, as it is a technique with a significant higher resolution. Besides the different shades of grey, there are also black spots on the electrode (MPL), which must be segmented separately in addition to the outline, the decoated area and the electrode. Looking at the histogram of the SEM image (Figure 6C), no clear separation between classes can be observed, and consequently segmentation following Otsu's method was unsuitable for SEM image processing. To achieve meaningful results with the simple threshold method for grey‐scale values in SEM images, the threshold for electrode, black spots and background needs to be adjusted manually for each SEM image to represent the respective area as accurately as possible.

Thresholding was only carried out once per image, as there is no variation of parameters in the automatic threshold setting. For the thresholding result of the SEM image, it is important to note that there are deviations depending on the manual threshold setting, but these were not shown or considered here.

#### 3.2.3 Trainable WEKA segmentation

Trainable WEKA Segmentation is a collection of machine learning algorithms that can perform pixel‐based segmentation,^38^ which is provided as a plugin for ImageJ. First, the algorithms are trained on predefined regions of the image, and then the rest of the image is segmented based on the trained model. The predefined regions are saved in the ROI Manager (ImageJ) for better repeatability. The feature extraction algorithms have to be adapted to the selected images and classes of interest. For SEM images, the default image features were chosen (Gaussian blur, Hessian, Membrane projections, Sobel filter, Difference of Gaussians).^38^ No feature selection was required for lower resolution µXRF images. As classifier always the default FastRandomForest was used. Since the result is highly dependent on the user‐specified regions, the images were segmented repeatedly with different specified regions as training data. The representative areas for the segmentation classes were also marked by freehand area selection or freehand lines. Only seemingly reasonable WEKA segmentations were accepted, clearly wrong segmentations were reworked or other areas were selected as user‐specified regions. This was used to calculate the decoating efficiency three to five times for each image to ensure reproducibility and to estimate the potential scattering.

**FIGURE 8:**
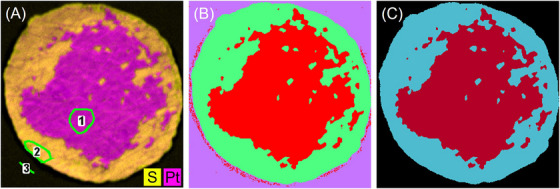
(A) µXRF image marked with predefined areas for WEKA training (1—electrode; 2—decoated area; 3—background); (B) WEKA result; (C) false colour image (red—electrode; blue—decoated membrane).

Figure 8 shows the original µXRF image with the predefined areas for WEKA training (Figure 8A), the resulting image from WEKA segmentation (Figure 8B) and the false colour image (Figure 8C). The step from the resulting image from WEKA to the final false colour image was again implemented with an in‐house developed Python code, where everything outside the membrane outline is defined as background. This allows the elimination of incorrectly segmented pixels, as visible in Figure 8B.

## 4 RESULTS AND DISCUSSION

### 4.1 Imaging

#### 4.1.1 SEM‐BSE

**FIGURE 9:**
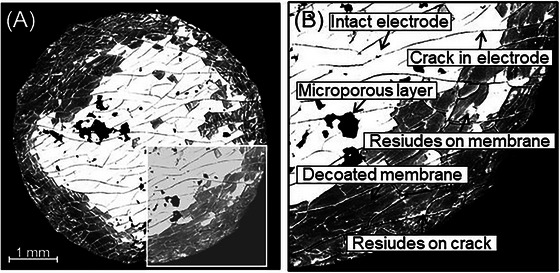
(A) SEM‐BSE image of sample 5000/1. (B) Detailed view on characteristic features in SEM‐BSE image.

The SEM images with a pixel size of 3.5 µm show the surface structures of the exposed layers in detail (Figure 9A). In addition to the decoated area in grey and the remaining electrode coating material in white, black spots can be seen on the electrode, indicating the carbon‐containing microporous layer which still remains on the electrode after stressing. Therefore, the black areas are considered to be still coated. Dark lines caused by cracks in the electrode can also be seen in the white, that is, still coated, areas. These cracks have already occurred during the manufacture of the CCM before use or during operation.^39^ All relevant features are highlighted in Figure 9B.

Apart from the clearly coated parts (white areas and dark spots in Figure 9B), there are additional bright areas visible as scattered, small‐size electrode residues within the predominantly decoated area. These are caused by thin electrode residues remaining on the membrane that are as bright as the intact electrode, even though the electrode is no longer at its full electrode thickness. Residues also remain on the cracks in the electrode and show high reflection signals with the BSE detector in the SEM. Both features can lead to underestimating decoating efficiency.

Another influencing factor in image acquisition is the contrast. Contrast determines whether the outline of the F‐ and S‐containing membrane is visible or how much the highly reflective platinum group metals shine. A high brightness gradient across the SEM image can also make these images difficult to evaluate, as in this case the electrode and the decoated membrane may have the same grey value. The method determines not only the surface material, but also the structures, and the resulting grey values can vary with the topography of the sample.

#### 4.1.2 µXRF

**FIGURE 10:**
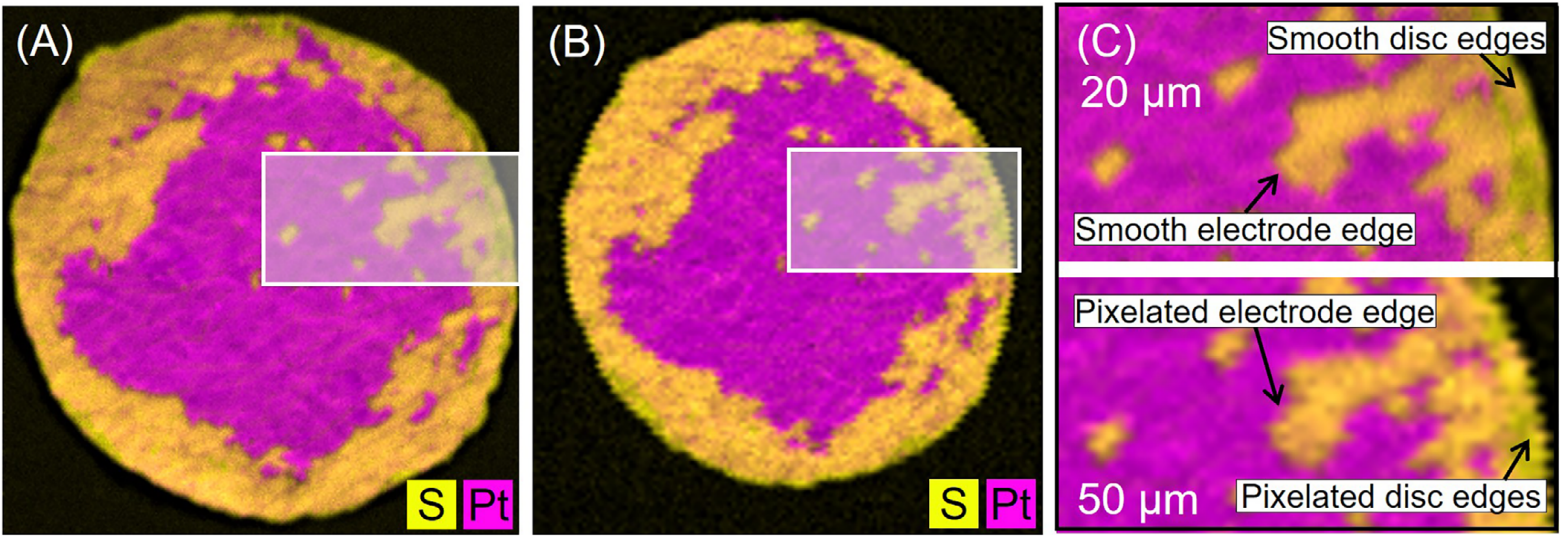
(A) Merged µXRF map with 20 µm spot size of sample 5000/1. (B) Merged µXRF map with 50 µm spot size of sample 5000/1. (C) Detailed view on characteristic features in µXRF maps of (A) at top and (B) at bottom.

For µXRF, the imaging results in Figure 10 illustrate the influence of spatial resolution for both selected pixel sizes, 20 µm (µXRF20) and 50 µm (µXRF50) (Figure 10A and B, respectively). Despite the less smooth appearance of boundaries for 50 µm resolution (e.g., pixelated disc edges), overall the same areas could be detected at similar extends. A detailed comparison of the two resolutions is shown in Figure 10C. Due to the roughly 10 times larger spot sizes compared to SEM, the depth of detail is lower than with the SEM images for both spot sizes, which means, for example, that the cracks in the electrode are no longer shown in such detail. This, on the other hand, reduces the number of features to only material boundaries without structural surface features and, thus, simplifies the processing and evaluation of the image data.

Compared to the images from SEM‐BSE, the resulting merged Pt‐S‐based µXRF map is not able to separately detect the MPL‐spots remaining on top of the electrode, which are associated with still coated areas. Using Pt as an indicator, results in a merged area of electrode plus MPL. Furthermore, since the X‐ray penetrates the materials to a certain extent, Pt signals from the backside of the coating also reach the detector. Figure 4A clearly shows the electrode of the front side in light grey and that of the rear side in dark grey. This can be explained by the relatively large information depth estimated for Pt in this study (∼379 µm) in comparison to the membrane thickness (40 µm). Due to the small membrane thickness compared to the information depth and the same Pt catalyst on both sides, the distinction between the two electrode sides must be considered when interpreting data in this specific application. This can be done by using the S‐map, which clearly shows where the membrane has been exposed by comminution (see Figure 4B). Therefore, both sides of a CCM must always be analysed in order to clearly determine the success of decoating for both electrodes.

#### 4.1.3 EDS

**FIGURE 11:**
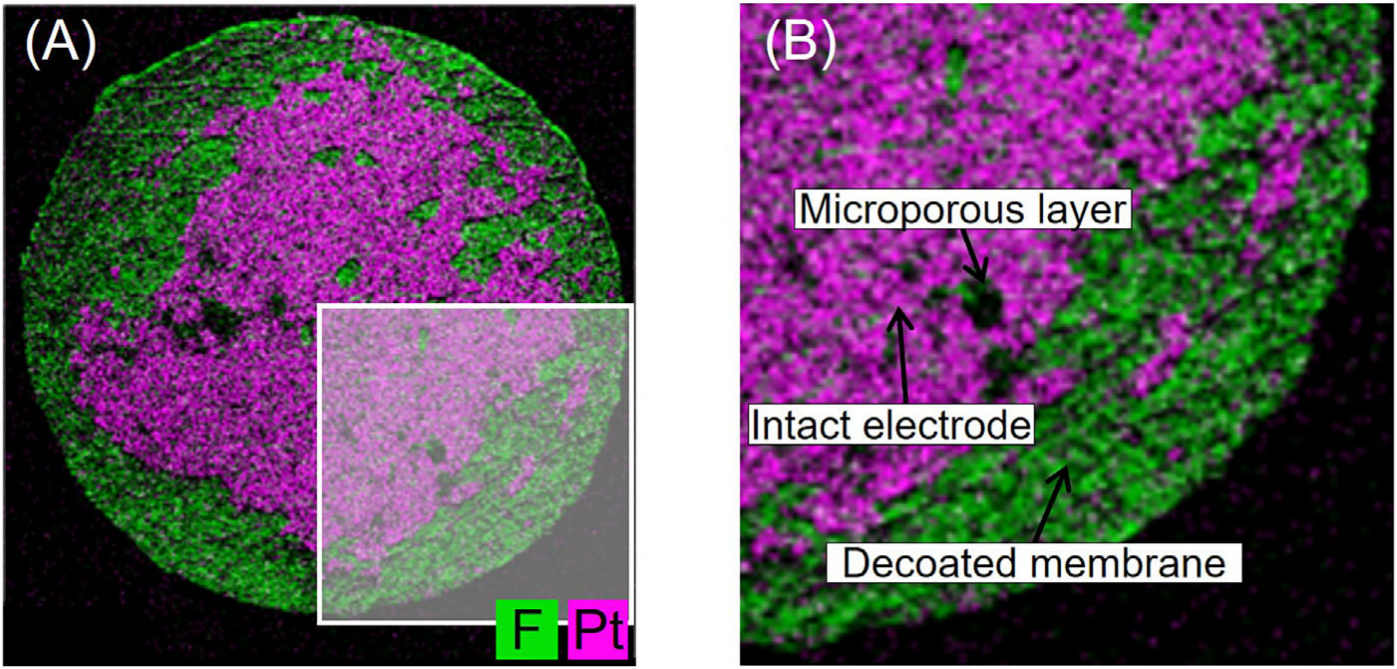
(A) Merged and coloured EDS map of Pt and F of sample 5000/1. (B) Detailed view on characteristic features in EDS map.

As with µXRF, EDS can be used to record elemental maps and, thus, detect decoated areas. The pixel size of the elemental maps amounts to around 20 µm, which is consistent with the pixel size of the µXRF20 images. A recoloured and merged image of the EDS elemental maps (Pt and F) is shown in Figure 11A. Another difference with µXRF is that the areas of carbon‐containing MPL show neither Pt nor F on the elemental distribution images due to the smaller penetration depth of the electron beam. While the electron beam of EDS penetrates only a few micrometres and does not reach the electrode, the X‐ray beam of µXRF can penetrate up to several hundred microns, retrieving information from the opposite side of the CCM. Therefore, the elemental map deriving from EDS shows black spots where MPL covers the electrode and blocks the electron beam (see Figure 11B), like in the SEM images. In comparison to the other two methods, it is evident that the areas under consideration are not distinctly delineated from one another. Instead, they are exhibited in a more scattered manner.

In general, decoating efficiency can also be determined from EDS elemental maps, but this analysis method has a long measurement time, and the parameters of the imaging analysis have to be further adjusted for the purpose of determining the decoating efficiency. In order to achieve meaningful decoating efficiencies, it would also be necessary to adapt the procedure for thresholding and WEKA to the scattered appearance. It may be necessary to carry out additional image processing, such as smoothing, prior to the subsequent procedure.

Therefore, the EDS analysis will not be further considered in this study, although the measurement method itself in principle is suitable for estimating decoating efficiency and can be optimised for future application.

### 4.2 Determination of decoating efficiency

**FIGURE 12:**
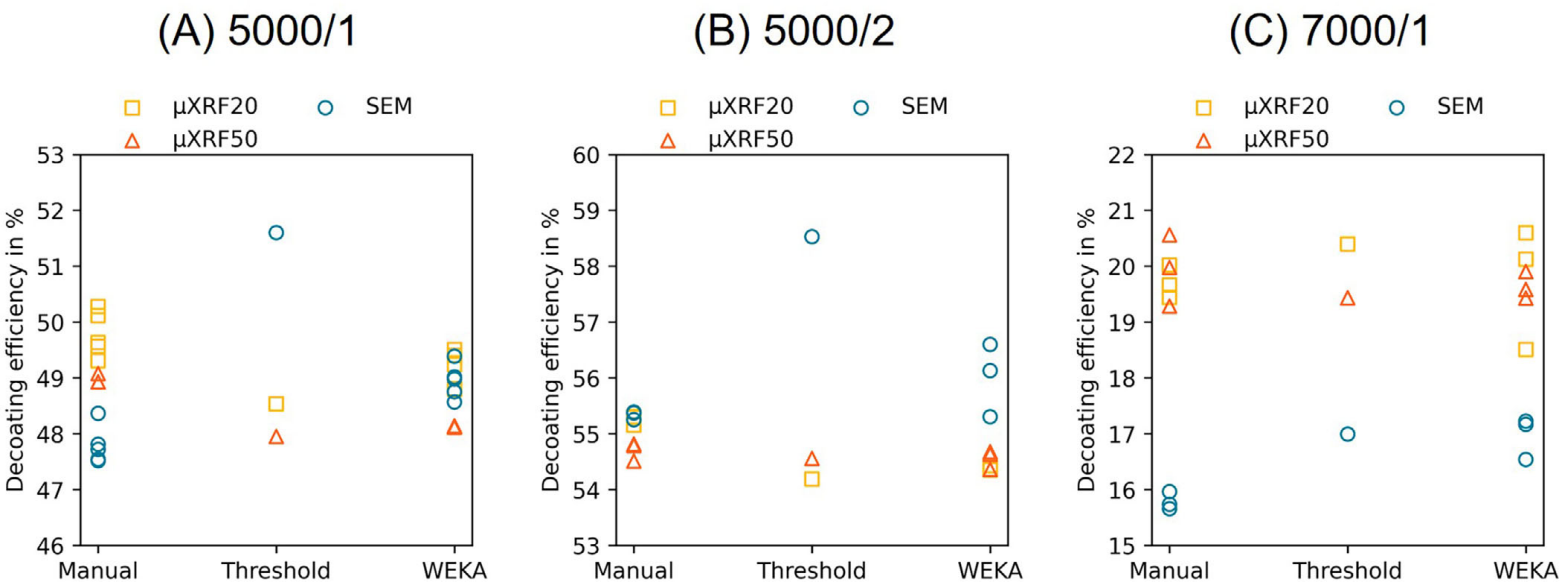
Decoating efficiency per imaging technique over segmentation method for sample (A) 5000/1; (B) 5000/2; (C) 7000/1.

Following the selection of the optimal image pre‐processing workflow, the decoating efficiency can be determined as described in Section 3.2. The decoating efficiency was calculated five times for each image acquired by individual imaging methods (SEM, µXRF20, µXRF50), which underwent segmentation by the three different methods (Manual, Threshold, WEKA). The result is a plot of the decoating efficiency over the segmentation methods, colour‐coded according to the imaging methods (yellow squares for µXRF20, orange triangles for µXRF50 and blue circles for SEM). Figure 12 shows one diagram for each membrane sample analysed.

Interpreting Figure 12A from an imaging method‐specific point of view, the maximum variation in decoating efficiency for one image processed by the same segmentation method, except for threshold, is about 1 percentage point (%pt.), that is, having a standard deviation of about 0.3%pt. This indicates that the segmentation methods themselves provide reproducible results for the decoating efficiency within a range of ± 0.5%pt. When comparing different imaging methods, a range of ∼4%pt. on the estimated decoating efficiencies can be observed. This shows a high precision (hence reproducibility) for decoating efficiency calculations when using specific combinations of imaging and segmentation methods; however, this is highly dependent on the imaging method chosen. A similar result can be seen when looking at another sample (Figure 12B). The total scatter range for estimated decoating results over all imaging methods is ∼4.5%pt., being particularly high for the thresholding segmentation method, whilst the standard deviation of decoating for each segmentation method is slightly lower than in Figure 12A at about 0.1%pt. (except for SEM‐WEKA).

**FIGURE 13:**
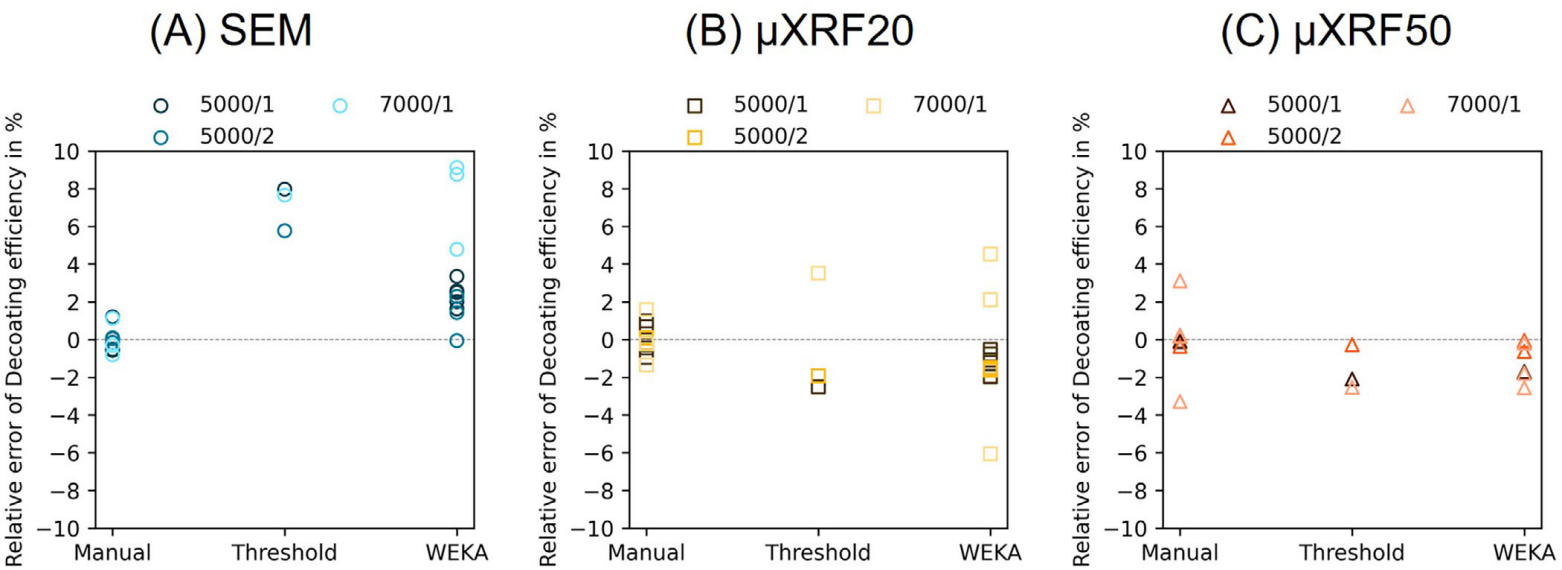
Decoating efficiency error relative to manual method for (A) SEM; (B) µXRF20; (C) µXRF50.

**FIGURE 14:**
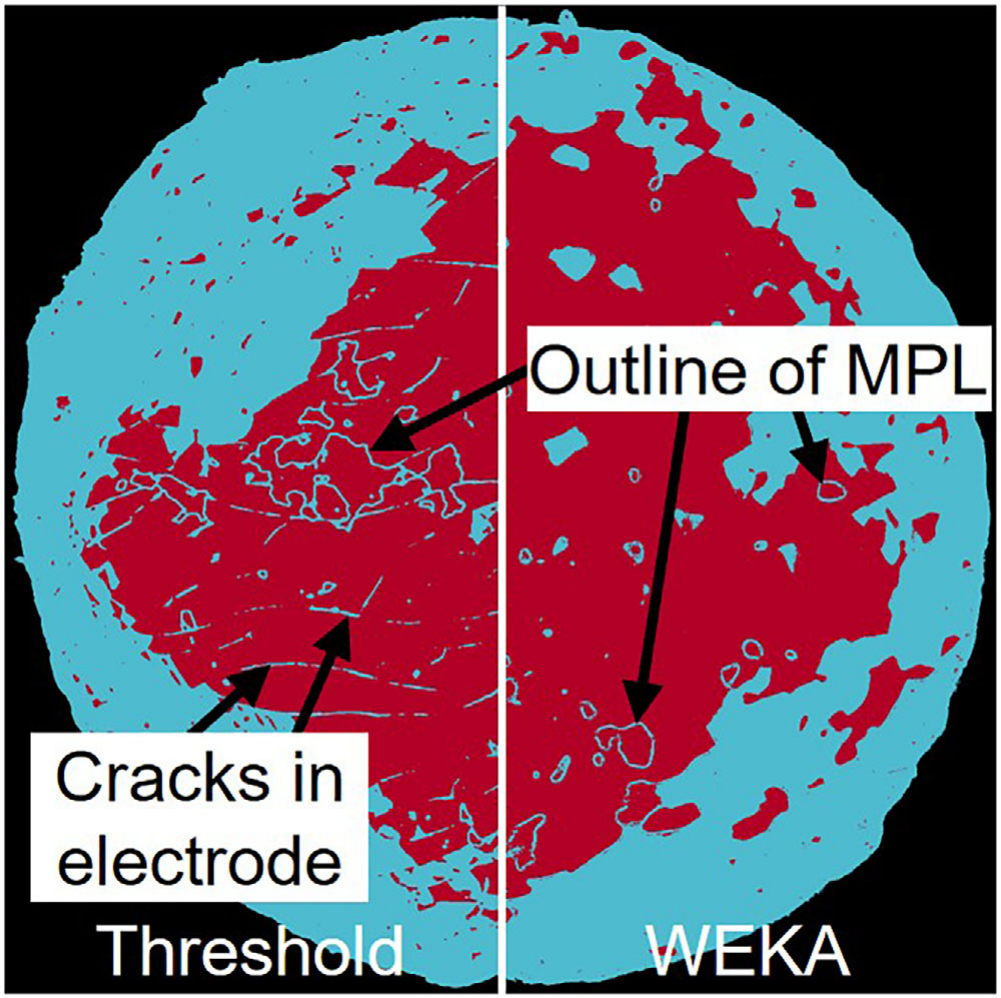
False colour image of sample 5000/1 based on SEM image (Figure 8), left processed with threshold, right processed with WEKA (red—electrode; blue—decoated membrane).

To better understand the reasons for the variation observed in decoating efficiency estimations, the results for each analysis method are presented separately as a relative error to the mean of the manual method, which is defined as reference. With this plot, the different imaging and segmentation combinations can be analysed across different samples. Figure 13A shows the relative error of the decoating efficiency for the SEM images for three samples. It can be clearly seen that both threshold and WEKA overestimate the decoating efficiency compared to the manual segmentation. This is due to the presence of cracks in the electrode and MPL, which leads to misinterpretation and misclassification (see Figure 14). Areas that are actually still coated (cracks and outlines of MPL) are assumed to be decoated, as the grey value of these features corresponds locally to that of the decoated membrane. The decoating efficiency is therefore higher than the reference value. Referring to Figure 13A, the threshold method deviates by at least 6% and up to 8% from the mean value of the manual method (cracks and MPL are misinterpreted), while WEKA can lead to significantly smaller deviations from 0 to 3.3% for 5000/1 and 5000/2 (only MPL is misinterpreted).

In general, SEM images can be used to determine decoating efficiency. However, a thresholding method alone is not suitable for evaluation due to the many different shades of grey in the images. The high‐resolution imaging of surface structures, which also captures the shadows of the membrane surface in a monochromatic grey‐scale data set, is a source of misclassification. The grey value is not only related to the material but also to the structures. With WEKA, the evaluation can be better adapted to the sample‐specific features through targeted training. However, it should be noted that some features still may be misinterpreted.

**FIGURE 15:**
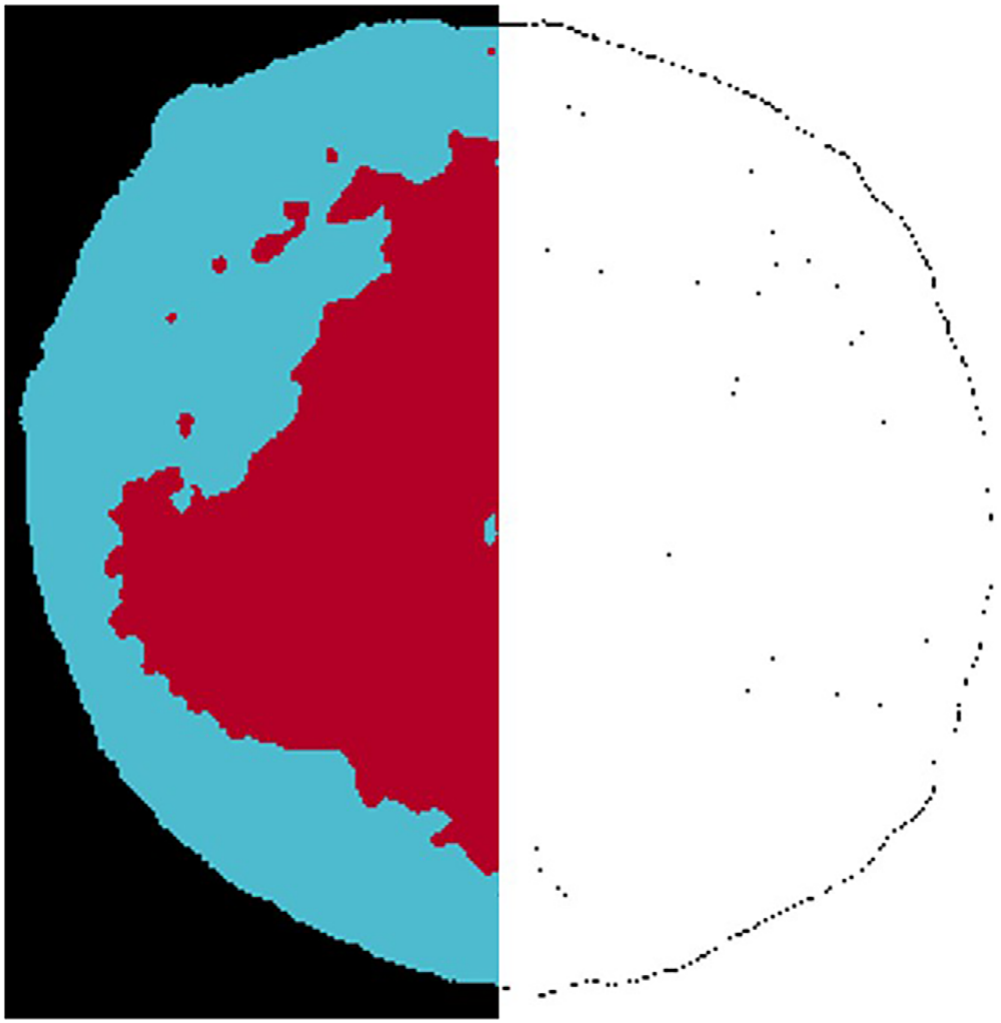
False colour image of sample 5000/1 based on µXRF20 (left), difference (black pixels) between a threshold false colour image and a WEKA false colour image (right) (red—electrode; blue—decoated membrane).

The consideration of the relative error for µXRF20 (Figure 13B) shows a clearly different picture than for SEM. The relative deviation from the mean manual value is less than 2.5% for threshold and WEKA. Only sample 7000/1 shows again higher deviations. Moreover, the decoating efficiency tends to be underestimated, a phenomenon that is also observed in µXRF50 with a larger spot size of 50 µm, which seems more robust also for the sample 7000/1 (Figure 13C). In addition, threshold and WEKA show a good performance in estimating decoating efficiencies for µXRF image data. The minor variations between the two methods are due to a difference of one maximum pixel in the outline of the sample and not to a different segmentation in the area of the electrodes. This is illustrated in Figure 15, which shows this difference (black pixels) between a threshold false‐colour image and a WEKA false‐colour image for sample 5000/1. Additional to the marginal differences between the techniques, the evaluation with the threshold method has the advantage that it can be done automatically with Python code. This eliminates the influence of the user in marking the predefined areas required by WEKA and speeds up the evaluation process.

**FIGURE 16:**
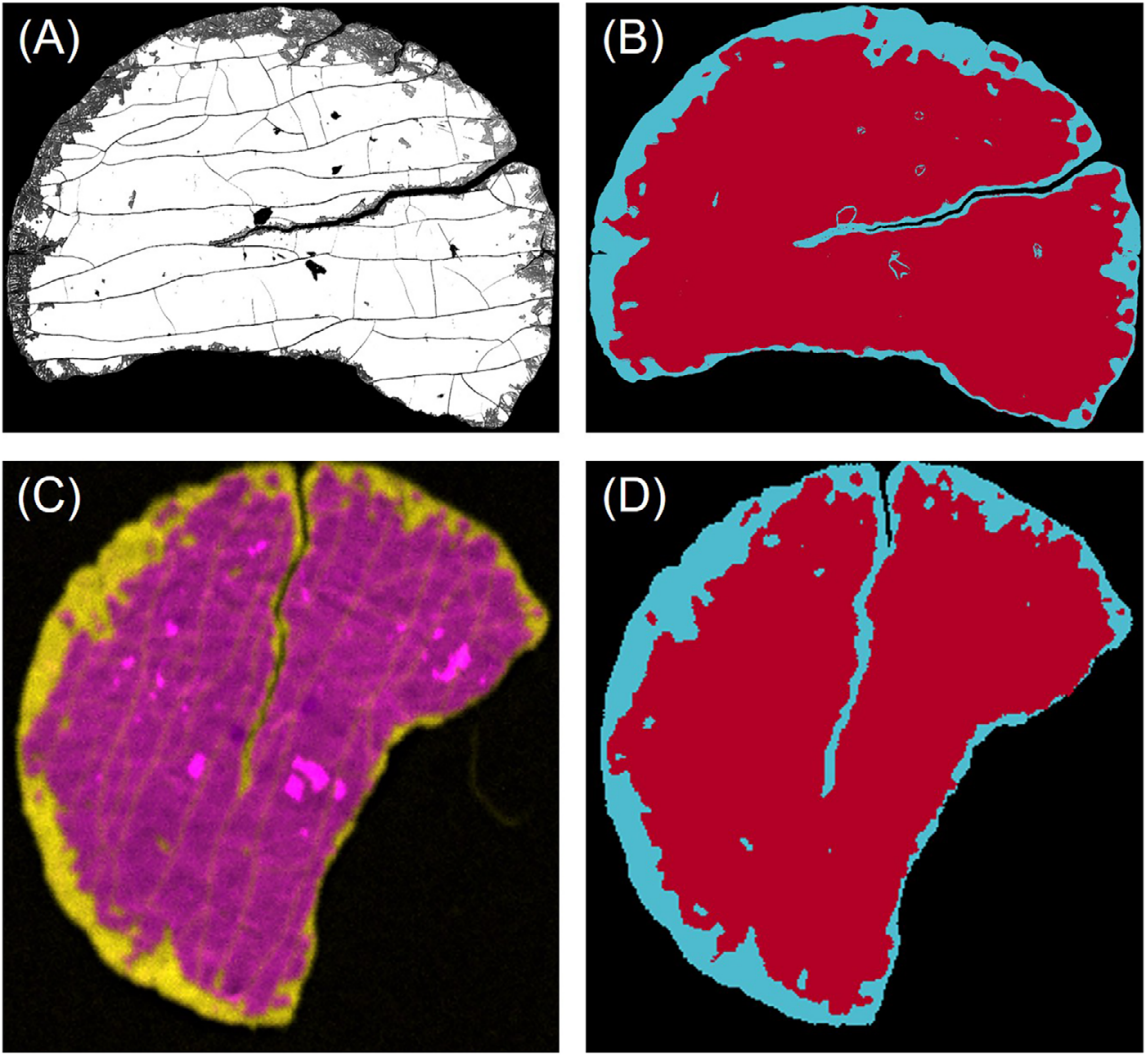
Images of sample 7000/1 (A) SEM and (B) related WEKA false colour image; (C) µXRF20 and (D) related WEKA false colour image (red—electrode; blue—decoated membrane).

As previously noted, decoating efficiencies estimated for sample 7000/1 show more deviations when compared to other samples. On the one hand, this is due to the appearance of the sample, since the sample was torn and partially fractured by a higher stress intensity in the milling process (7000 rpm), causing fractures in the membrane itself (see Figure 16). In addition, the anode side of the electrode has a finer fracture structure, while the cathode side has a coarser fracture structure. On the other hand, the differences in the imaging methods become clear, such as the variation in resolution and the associated level of detail of the images.

Figure 16 clearly illustrates that the long crack in sample 7000/1, in particular, is responsible for the of nearly 3%pt. difference in estimated decoating efficiency between SEM and µXRF evaluated with WEKA. The crack in the SEM image has a width of more than 20 pixels at the widest point and at least 3 pixels at the narrowest point. As a result, the black adhesive pad is visible as a background through the crack in the SEM image and it is segmented as such. This is not the case with µXRF maps because the pixel size of 20 or 50 µm blurs this crack which is only one or two pixels wide. This is why it is detected as a decoated area and not as background (see Figure 16B and D). Other problematic details are the tight decoated areas at the edge of the sample and the finer fracture structure, where clear segmentation is difficult. In addition, there are small cracks in the membrane on the SEM image that are sometimes detected as decoated and sometimes as background and are not visible on the µXRF maps. This can be explained by the 14 times higher resolution of the SEM, which displays an approximately 5 mm sample with 1.75 million pixels, as opposed to just under 10,000 pixels for the 50 µm resolution of the µXRF.

In summary, especially for samples with low decoating efficiency and fine coating or surface structures, but also for any other analytical task, the method selection must be made according to the application scenario. High‐resolution analysis, as with SEM combined with detailed pixel‐based segmentation, can promote scientific and process understanding, which is particularly necessary for the development of appropriate decoating processes for the different types of CCM. The efficient automatic detection of high or low decoating efficiency with µXRF combined with fast colour thresholding is a crucial aspect of industrial process evaluation. This can be used for standardised process comparisons and parameter studies.

One possible application of the methods described above is the determination of decoating efficiency as a function of stress intensity. As described in the Section 2, samples consisting of 10 punched out pieces of a PEMWE CCM were stressed at different speeds of the laboratory hammer mill. The stressed membrane pieces were analysed by µXRF at a pixel size of 50 µm from both sides and segmented by thresholding. As the samples consist of WE cell, Ir is used as an indicator for the electrode on the anode side. The rest of the procedure is identical to that described here.

**FIGURE 17:**
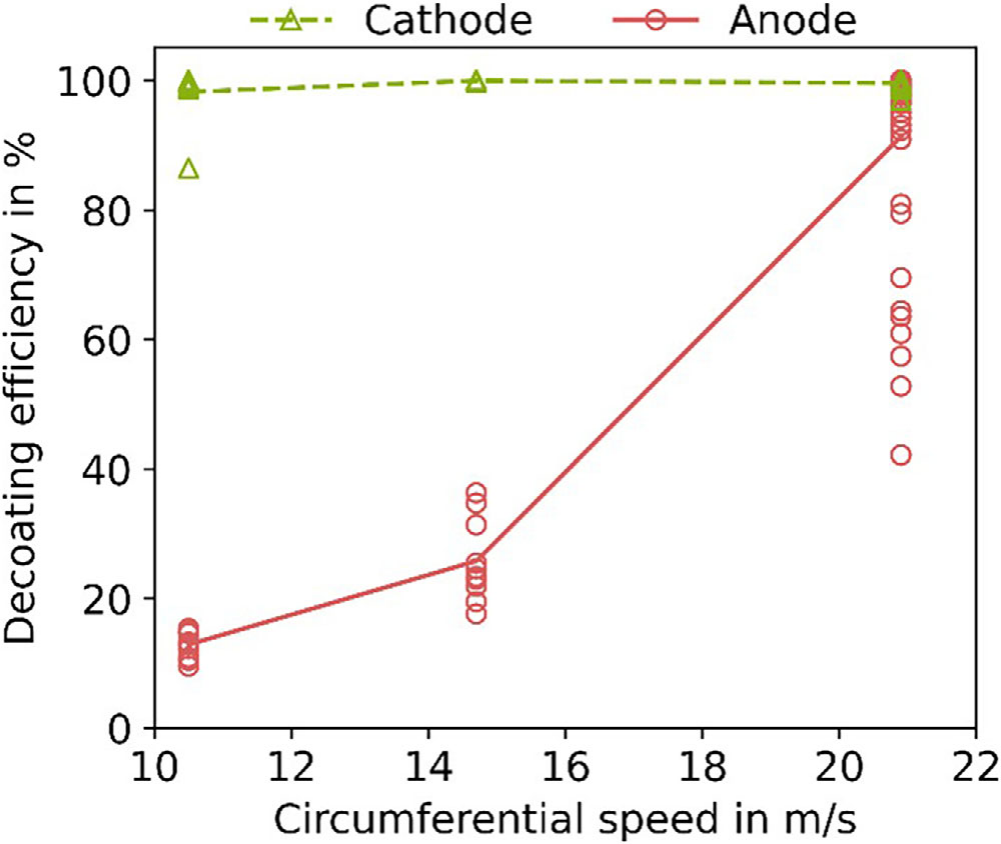
Decoating efficiency for anode and cathode of a PEMWE CCM with variation of the stress intensity; µXRF50 with colour thresholding was used for the determination.

Figure 17 shows the decoating efficiency for each individual particle for the cathode side in green triangles and for the anode side in red circles. The lines (anode dashed; cathode solid) represent the average of the individual values. The cathode side of the PEMWE CCM is almost completely decoated even at low stress intensity (mean value 98.3%), while the anode side is only 12.9% decoated, mainly at the outline. At the highest speed of 20.9 m/s, the anode is also 91.3% decoated. The result of the investigation with the proposed analysis method shows a selective decoating of the CCM, suggesting a possible enrichment of the cathode and anode product at different stress intensities.

## 5 CONCLUSION

The evaluation of mechanical decoating of PEM cells is possible using SEM‐based imaging or µXRF‐based element mapping methods. The subsequent image processing and the pixel size of the images with the resulting resolution of details can significantly affect the calculated decoating efficiency.

The SEM images are difficult to segment using automated thresholding methods due to the stronger scatter of grey values, which results from the high resolution of many detailed structures. This results in a relative error compared with manual segmentation of up to 8%. Also, an evaluation with WEKA can be difficult due to varying contrast and possible carbon residues on the electrode resulting in deviations just as high as with thresholding. However, SEM images in general are useful for showing details such as electrode residues on the membrane and cracks in the membrane, which help to understand the decoating process. EDS analysis can be used to obtain suitable elemental maps with detecting Pt and F to determine the decoating efficiency as well. For a reliable result, the measurement parameters must be adjusted. Also, due to the long measurement time, EDS is more suitable for smaller sections of a sample, for example, to detect residues on the membrane by elemental mapping.

The µXRF mapping shows significantly less structural details due to the spatial resolution of 20 and 50 µm respectively. Since WEKA and threshold usually provide results with similar relative errors in the range up to –2.5% for µXRF elemental maps, an automated evaluation with threshold method using Python can be applied. Furthermore, the measurement time can be shortened by a factor of six by selecting a larger spot size of 50 µm, which always leads to consistent results with a maximum relative error of –2.5%. In general, decoating efficiencies based on µXRF mappings can be specified with a standard deviation of 0.1 to 0.4%pt. for 20 µm and 0.1 to 0.2%pt. for 50 µm, whereas the results of the evaluation of SEM images should always be questioned. However, it must be noted that due to the higher penetration depth of the X‐rays in µXRF analysis, the back side of the sample is visible, which can influence the evaluation if the catalyst is the same on both anode and cathode, as is the case with PEMFC cells.

This work presented an analytical method to determine the decoating efficiency for stressed CCM samples of PEMC. For further application, the outcomes can be transferred from FC to WE cells. For µXRF, the transfer from to Ir as target element does not present a challenge as only Ir needs to be added to the evaluation for the modified catalyst in the anode. Furthermore, there are no changes to the SEM evaluation required, as Ir is also a highly reflective element like Pt. In addition, both methods are robust to variations in the thickness of the membrane between FC and WE. Thus, a method has been developed to determine the decoating success of samples for which neither the mass nor RGB images can be used.

Using the method described here, systematic decoating tests can be performed and evaluated on PEMWE cells of very small scale. The decoating efficiencies determined can then be used to identify the parameters for the most efficient mechanical decoating process. The target is to achieve the required recycling rate of 99% for the critical precious metals.

## AUTHOR CONTRIBUTIONS

Malena Staudacher: conceptualisation, methodology, investigation, writing—original draft, writing—review & editing. Andréa de Lima Ribeiro: investigation, writing—original draft, writing—review & editing. Ruben Wagner: investigation, writing—original draft, writing—review & editing. Margret Fuchs: writing—review & editing. Anja Weidner: writing—review & editing. Thomas Buchwald: writing—review & editing. Urs Peuker: funding acquisition, project administration, resources, writing—review & editing.

## CONFLICT OF INTEREST STATEMENT

The authors declare that they have no known competing financial interests or personal relationships that could have appeared to influence the work reported in this paper.

## DECLARATION OF GENERATIVE AI AND AI‐ASSISTED TECHNOLOGIES IN THE WRITING PROCESS

During the preparation of this work the authors used DeepL in order to enhance the readability of the text. After using these tools, the authors reviewed and edited the content as needed and take full responsibility for the content of the publication.
